# *Toxoplasma gondii* infection in pig intended for human consumption: seroprevalence, risk factors and influence of biosecurity measures

**DOI:** 10.1590/S1984-29612023060

**Published:** 2023-11-13

**Authors:** Agostinho Sergio Scofano, Igor Falco Arruda, Jessica Nogueira Teixeira, Nathalie Costa da Cunha, Elmiro Rosendo do Nascimento, Maria Regina Reis Amendoeira, Patrícia Riddell Millar

**Affiliations:** 1 Instituto de Defesa Agropecuária e Florestal – IDAF, Cariacica, ES, Brasil; 2 Programa de Pós-graduação em Higiene Veterinária e Processamento Tecnológico de Produtos de Origem Animal, Faculdade de Veterinária, Universidade Federal Fluminense – UFF, Niterói, RJ, Brasil; 3 Laboratório de Protozoologia, Instituto Oswaldo Cruz, Fundação Oswaldo Cruz – FIOCRUZ, Rio de Janeiro, RJ, Brasil; 4 Programa de Pós-graduação em Medicina Veterinária, Universidade Estadual Paulista “Júlio de Mesquita Filho” – UNESP, Botucatu, SP, Brasil; 5 Departamento de Saúde Coletiva Veterinária e Saúde Pública, Faculdade de Veterinária, Universidade Federal Fluminense – UFF, Niterói, RJ, Brasil; 6 Departamento de Microbiologia e Parasitologia, Instituto Biomédico, Universidade Federal Fluminense – UFF, Niterói, RJ, Brasil

**Keywords:** Toxoplasmosis, swine, zoonosis, public health, infectious diseases, Toxoplasmose, suíno, zoonose, saúde pública, doença infecciosa

## Abstract

A serologic and epidemiologic study was carried out in order to determinate herd and animal seroprevalence and associated factors for *Toxoplasma gondii* in commercial pigs from Espírito Santo state, Brazil. Blood samples were collected from 416 pigs from 55 producer farms in 27 municipalities. An indirect immunofluorescent assay (IFA) was performed to estimate the seroprevalence of *T. gondii* and identify the associated risk factors using a questionnaire. The *T. gondii* antibody prevalence rate in commercial swine herds was 15.4% (64/416) using a cutoff of 1:64. The seropositivity for *T. gondii* was related to the presence of cats, water origin and age of swine in the increase of seroprevalence, and the existence of internal isolation fences and use of composting chambers as protective factors. To the best of our knowledge, this is the first study to report anti- *T. gondii* antibodies in the serum of pigs in the state of Espírito Santo, Brazil. This finding is important to public health because seropositive pigs can harbor tissue cysts in their meat, thereby representing a zoonotic risk for consumers of raw or undercooked porcine meat or its products.

## Resumo

Um estudo sorológico e epidemiológico foi realizado com o objetivo de determinar a soroprevalência nas granjas e animais e também em fatores associados para *Toxoplasma gondii* em suínos comerciais do estado do Espírito Santo, Brasil. Foram coletadas amostras de sangue de 416 suínos de 55 granjas produtoras de 27 municípios. A reação de imunofluorescência indireta (RIFI) foi realizada com o uso de questionário, para estimar a soroprevalência do *T. gondii* e identificar os fatores de risco associados. A taxa de prevalência de anticorpos contra *T. gondii,* nos rebanhos comerciais de suínos, foi de 15,4% (64/416) com ponto de corte de 1:64. A soropositividade para *T. gondii* foi relacionada à presença de gatos, origem da água, a idade dos suínos no aumento da soroprevalência, a existência de cercas de isolamento interno e uso de câmaras de compostagem como fatores de proteção. De acordo com este estudo, ele é o primeiro a relatar anticorpos anti-*T. gondii* no soro de suínos no estado do Espírito Santo, Brasil. Este achado é importante para a saúde pública, pois suínos soropositivos podem abrigar cistos teciduais em suas carnes, representando um risco zoonótico para os consumidores de carne suína crua ou mal cozida ou seus produtos.

## Introduction

Zoonoses are infectious diseases that occur when humans interact with infected animals, their products or environment, and are of viral, bacterial, or parasitic origin. Among foodborne parasitic diseases, toxoplasmosis, caused by *Toxoplasma gondii*, is highlighted (Rossi et al., 2014). Toxoplasmosis is a zoonosis of considerable concern to public health (Arruda et al., 2021) and is highly prevalent in Brazil. *T. gondii* is an obligate intracellular protozoan capable of infecting mammals and birds. In humans, the infection occurs mainly by ingestion of raw or undercooked meat containing tissue cysts, or, food or water contaminated with sporulated oocysts, or via congenital transmission (Almeria & Dubey, 2021; Moura et al., 2013). Toxoplasmosis is conventionally associated with cats, the only definitive host of the parasite capable of eliminating oocysts; however, in humans, approximately 50% of cases are foodborne (Herrero et al., 2016).

In Espírito Santo State, serological studies have demonstrated the presence of *T. gondii* in sheep (Acosta et al., 2021; Tesolini et al., 2012), chicken (Beltrame et al., 2012; Ferreira et al., 2018; Pena et al., 2013), cats (Fux et al., 2020), and dogs (Acosta et al., 2016). The prevalence of the disease in humans has also been described (Abreu et al., 1998; Areal & Miranda, 2008; Sessa et al., 1980). According to Balbino et al. (2022), two toxoplasmosis outbreaks have been reported in the state. The first was in 2014 in the city of Serra, affecting 130 people; however, the source of infection was not discovered. The second was in 2019 in the municipality of Rio Bananal, affecting 17 children from the same day care center; the Department of Health of Espírito Santo reported that the outbreak was associated with the ingestion of oocysts present in sand and soil. Despite the ubiquitous presence of the parasite and its proven circulation among humans, animals, and the environment, few studies on toxoplasmosis and its epidemiology have been reported from the state of Espírito Santo. Further, although the presence of *T. gondii* in pigs is widespread in Brazil (Dubey et al., 2020), related studies have not been reported from this region.

Among livestock, serological surveys usually indicated the incidence of *T. gondii* infection in the swine population, although the prevalence varied according to the age of pigs, size of the pig herd sampled, facilities on the farms, management of sanitary facilities, presence of rodents, and presence of favorable temperature for oocysts sporulation (Herrero et al., 2016; Oliveira et al., 2018). In the past decade, *T. gondii* DNA has been detected in processed pork or uncured pork products, rendering the consumption of raw and undercooked pork an important way of toxoplasmosis transmission to humans (Dubey et al., 2020; Papatsiros et al., 2021). The Brazilian pork industry increased its meat production from 3.3 million tons in 2011 to 4.7 million tons in 2021, of which 1.13 million tons were exported, ranking Brazil fourth among countries exporting pork products in 2021, with the domestic consumption reaching 16.7 kilos per capita (ABPA, 2022). These data demonstrate the importance of pig farming in Brazil.

The aim of this study was to evaluate the presence of anti-*T. gondii* antibodies in finishing swine raised for human consumption on different farms in Espírito Santo State, Brazil, and to assess the risk factors associated with the infection.

## Material and Methods

### Study design

**Figure 1:**
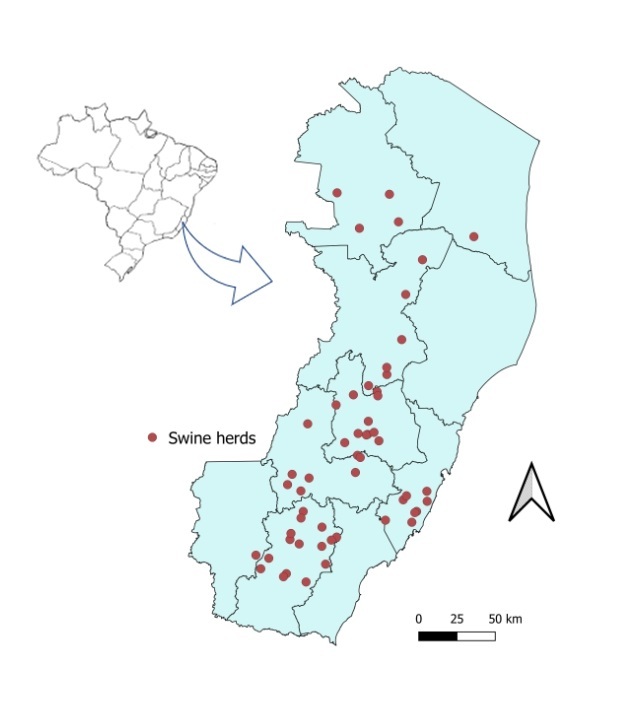
Distribution of swine population in farms participating in the study in the state of Espírito Santo, Brazil.

This was a cross-sectional analytical study performed on pigs raised for human consumption on farms in Espírito Santo State, Brazil (Figure 1).

A total of 55 farms, of producers who agreed to participate in the study, were selected after conducting an initial survey of the register of commercial pig farms in the Agricultural Integration System of the Institute of Animal and Forestry Defense of the State of Espírito Santo. These represented 72.4% of the total number of registered commercial farms.

To determine a representative sample, the total population of pigs, estimated as 241.550, was used as a reference (Brasil, 2017). Statistical analysis, based on the Epi Info 7.2.5 (U.S. Centers for Disease Control and Prevention, Atlanta, USA), determined the blood collection of 384 animals using an estimated prevalence of 50%, a 95% confidence interval (CI), and a maximum error of 5%.

A total of 416 blood samples, from 3-10 pigs per farm, were obtained. Owing to the difference in herd quantity between farms, proportional stratified sampling was performed. To meet the inclusion criteria for animal selection, discarded sows, those in the middle stage of pregnancy, and those in the final phase of maternity were selected. In finishing farms, pigs ready for slaughter were selected. Underweight animals or those undergoing drug treatments were excluded.

The division of the State into micro-regions was used according to the characteristics of their municipalities and for a better strategic planning of future actions.

The owners of the animals signed a consent and clarification form to participate in the research after being informed of the research objectives, confidentiality of their identities, and their right to refuse to participate at any time.

### Epidemiological questionnaire

To identify possible risk factors for infections in the pigs, an epidemiological questionnaire was provided to producers according to Silva et al. (2019) and Brazilian regulation (Brasil, 2017); it comprised variables such as sex, age, use of cats as rodent controls, presence of rodents, presence of an inner fence on the property, presence of composting chamber, type of water source (stream or well), use of chlorinated water for animals, and presence of reproductive disorders in sows.

### Risk classification of farms

A scoring system was assigned for each item of the questionnaire, following the pattern of Brazilian regulation (Brasil, 2002), with the addition of additional biosecurity variables. Each variable presented a score stipulated according to its application on the farm, and the sum of the scored points as well as the framework for the vulnerability of each farm were determined. Higher the number of points presented by the farm, greater the vulnerability of the system, with the vulnerability of the farms classified as high, moderate, low, or not vulnerable (well-protected).

### Sample collection

Pigs were randomly selected from the herd for blood collection. Blood samples were collected in 10 mL tubes that without anticoagulants. After clot formation the tubes were centrifugated (Bia 4000 centrifuge, Daiki) at 600 *× g* for 10 min to separate the serum. The sera were then transferred to sterile vials and stored at –20 °C until further analysis.

### Detection of anti-*T. gondii* antibodies

Samples were analyzed using the indirect immunofluorescence antibody test, following the procedure reported by Camargo (1964) and in accordance with the protocol established by Toxoplasmosis and Other Protozoan Diseases Laboratory (LabTOXO). Tachyzoites of the RH strain of *T. gondii* maintained in Swiss Webster mice were used as antigens. Positive and negative controls previously tested and stored in the serum bank of LabTOXO were used. Commercial conjugate anti-pig IgG FITC (Sigma-Aldrich) was used in all reactions at a dilution of 1:75 in Evans blue (Sigma-Aldrich). Samples were evaluated by a single technician that considered positive when the formation of the antigen-antibody-fluorescein conjugate complex was detected at a dilution of 1:64 or higher.

### Statistical analyses

Data of the serological experiments and epidemiological variables were analyzed using the Jamovi statistical V2.2.5 software. The Fisher’s exact test was used to compare the prevalence between microregions. Associations between two categorical variables were determined using Pearson’s chi-square test (χ2) or Fisher’s exact test, at a significance level of 5%. To evaluate the impact among the variables, odds ratios (OR) were described along with their respective 95% CI.

Multivariate analysis, including associated risk factors, was performed using a stepwise logistic regression model. Non-significant variables were sequentially removed. The model was run in Jamovi software until all remaining variables presented statistically significant values (*P* < 0.05).

## Results

Of the total samples collected, 79.8% (332/416) were from sows and the remaining were from piglets ready for slaughter on finishing farms. The *T. gondii* antibody prevalence rate in Espírito Santo, Brazil was 15.4% (64/416), with at least one animal showing a positive result in 56.36% (31/55) of the commercial farms studied. Most antibodies titers were 1:64 (61/416) with only 3 samples showing levels of 1:256. The prevalence of infection distributed by region in Espírito Santo is presented in Table 1.

**Table 1:** Region-wise distribution of *T. gondii* infection in naturally-infected pigs in Espírito Santo state, Brazil.

**Microregion**	**Sample Size**	**Number of positive animals**	**Prevalence (%)**	
			**in microregion** *	**general** **
**Metropolitana**	68	6	8.82^a^	1.44
**Central Serrana**	69	22	31.88^b^	5.25
**Sudoeste Serrana**	57	12	21.05^b^	2.88
**Central Sul**	153	12	7.84^a^	2.88
**Caparaó**	10	4	40.0^b^	0.96
**Centro Oeste**	30	6	20.0^a.b^	1.44
**Nordeste**	2	1	50.0^a.b^	0.24
**Noroeste**	27	1	3.7^a^	0.24

* Different letters (a,b) in the columm indicate significant differences (p<0.05);

** In general prevalence total samples (416) was used as denominator.

**Figure 2:**
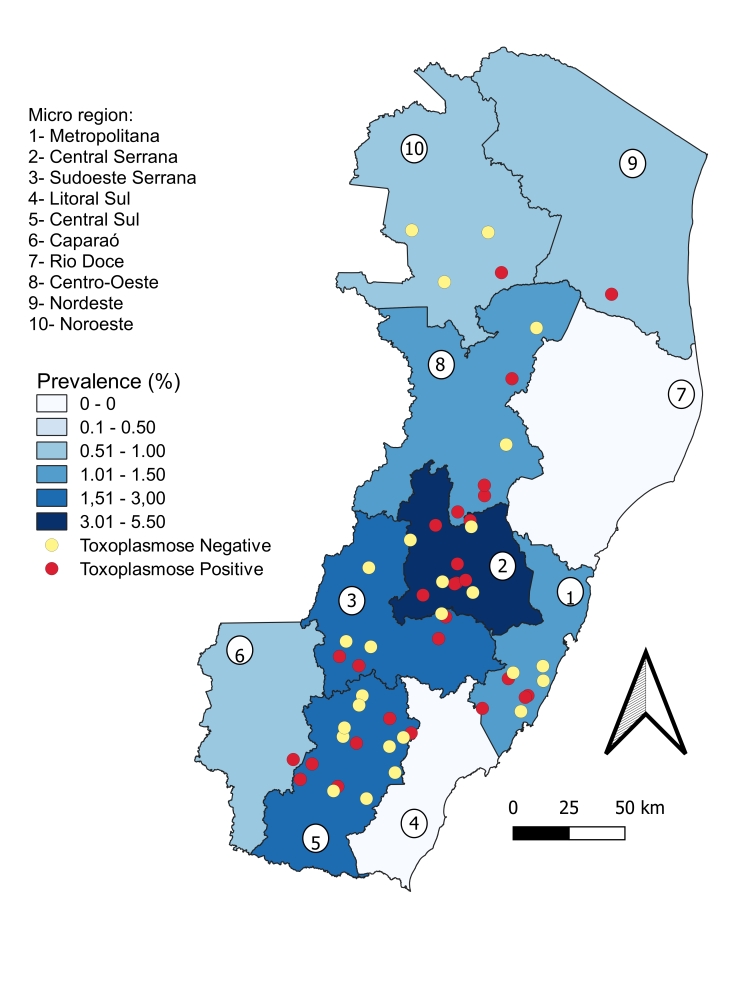
Geographic distribution of the 55 farms in the state of Espírito Santo, Brazil sampled in the study and their positivity status of anti-*Toxoplasma gondii* antibodies.

The geographic distribution of the 55 farms sampled in Espírito Santo State and their positivity status for anti-*T. gondii* antibodies are shown in Figure 2. Positive cases were dispersed among the municipalities studied but were more concentrated in the Central Serrana and Sudoeste Serrana micro-regions. These micro-regions were also those with the highest concentration of farms classified as having a moderate or highly vulnerable biosecurity level.

Eight properties were identified with positive status in more than 50% of the samples collected, and one with 100% positive samples (3/3). Just one property classified as low vulnerability showed a positivity of 50% in the collected sows. Only two farms were classified as moderate and the others presented a high vulnerability standard as biosecurity classification. The farm with 100% seropositivity was a small family swine farm and has been considered as highly vulnerable. One of the moderate vulnerable farms only performs the finishing phase and presented a seropositivity of 80% among the collected samples.

**Figure 3:**
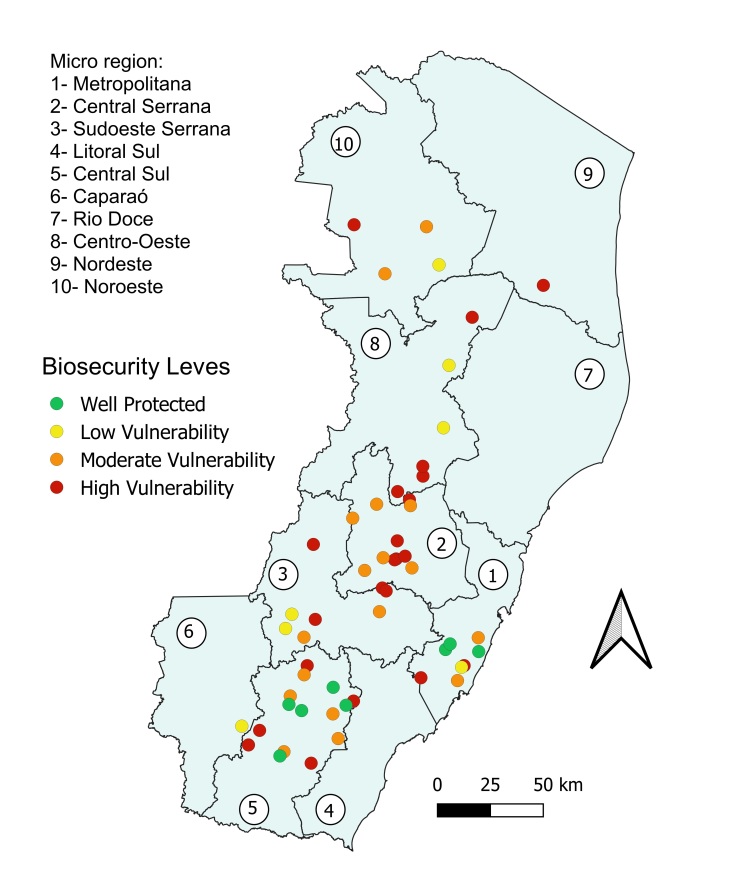
*T. gondii* infection risk map with respect to farm biosecurity in Espírito Santo State, Brazil.

Based on the biosecurity practices of the properties with positive cases, the vulnerability status of fifteen properties was classified as high, five were classified as moderate, five as low, and six as not vulnerable (well protected) (Figure 3). The Central Serrana micro-region was found to comprise farms classified only as moderate or high vulnerability. Among the properties with more than 50% positive cases, the vulnerability of four was classified as high, which included one with 100% positive cases.

Seropositivity for *T. gondii* was found to be statistically related (*P* < 0.05) to the following factors: presence of cats (OR 2.5; IC 1.39-4.48; 95%), water source (OR 3.51; CI 1.90-6.47; 95%) and age of pigs (OR 2.75; CI 1.14-6.62; 95%) can increase the seroprevalence rate, thus presence of internal insulation fence (OR 0.45; CI 0.26-0.79; 95%) and use of compost chamber on the farm (OR 0.43, CI 0.25-0.74, 95%) were classified as protective factors. In contrast, seropositivity was not statistically related (*P* > 0.05) to the presence of rodents, use of chlorinated water by animals, or reproductive disorders in the sows (Table 2).

**Table 2:** Risk factors associated with seroprevalence of *T. gondii* infection in pigs from Espírito Santo State, Brazil.

**Variable**		**Nº**	**% IFAT**	***p* Value**	**OR (CI 95%)**
**Use of cats for rodent control**					
	Yes	224	20.5	0.002	2.5 (1.4 - 4.5)
	No	192	9.4		
**Presence of rodents**					
	Yes	259	16.6	0.377	
	No	157	13.4		
**Inner Fence**					
	Yes	224	10.7	0.004	0.4 (0.2-0.8)
	No	192	20.8		
**Composting chamber**					
	Yes	289	11.7	0.002	0.4 (0.2-0.8)
	No	127	23.6		
**Water source**					
	Stream	64	32.8	<0.001	3.5 (1.9-6.5)
	Well	352	12.2		
**Use of treated water**					
	Yes	209	12.9	0.161	
	No	207	17.9		
**Reproductive disordes in sows**					
	Yes	161	14.3	0.622	
	No	255	16.1		
**Age Classification**					
	Sow	332	17.5	0.019	2.75 (1.1-6.6)
	Fattening pigs	84	7.1		

Nº: number of swines; IFAT: immunofluorescence antibody test; *p* Value: associations assessed by Pearson’s Chi-square (p<0,05); OR: odds ratio; CI: confidence interval.

Logistic regression analysis demonstrated that the following variables were statistically associated (*P* < 0.05) with *T. gondii* infection: composting chamber use (OR 0.4; CI 0.22-0.78; 95%), age of pigs (OR 2.62; CI 1.02-6.69; 95%) and water source (OR 4.64; CI 2.40-8.94; 95%).

## Discussion

The *T. gondii* antibody prevalence rate for farms in Espírito Santo State was 15.4%, which is similar to the averages commonly reported for comercial farms in Brazil (Feitosa et al., 2017; Lopes et al., 2021; Samico Fernandes et al., 2012). In general, the prevalence of *T. gondii* infection in comercial farms is lower than that in small farms because of better animal husbandry systems used (Bezerra et al., 2009; Gazzonis et al., 2018). To our knowledge, this is the first report on the prevalence of *T. gondii* in swine in Espírito Santo State, Brazil. These data are of public health relevance, because consumption of undercooked or raw meat of farm animals is a known major risk factor for *T. gondii* infection in humans; further, among food obtained from livestock species, pork is considered one of the main sources of infection, as reported in other studies (Dubey et al., 2020; Feitosa et al., 2017; Millar et al., 2008; Silva et al., 2021).

Regarding the biosecurity practices of the properties, the majority were classified as high vulnerable in this study. However, the result was not statistically significant (p>0,05). In contrast, Gazzonis et al. (2018) reported a significant difference, concluding that the risk of infection on farms is inversely proportional to the biosecurity practices.

Domestic cats are often used to control rodents on pig farms. In this study, seropositivity for *T. gondii* was found significantly related to the presence of cats used for rodent control, mainly due to deposition of oocysts into the soil or water, leading to a high level of environmental contamination. The presence of cats has been reported as a risk factor by other studies (Ortega-Pacheco et al., 2011; Sousa et al., 2014). However, Ortega-Pacheco et al. (2011) reported that *T. gondii* infection spreads rapidly in pig farms in endemic areas, regardless of the density of cats on the farm.

In this study, animals on farms that obtain water from streams showed a higher seropositivity for *T. gondii* than animals on those that used water from wells. Water contaminated is one of the main sources of infection in pigs (Dubey, 2009). It is possible that water from streams may be more contaminated with sporulated oocysts than well water. Further, it can be speculated that felines in the vicinity of such water sources could get infected with sporulated oocysts. However, the use of treated water by the animals did not result in a statistically significant difference in this study.

Age of the animal is also an important risk factor in toxoplasmosis because *T. gondii* seroprevalence increases with the animal’s age, possibly due to longer exposure to the parasite (Dubey et al., 2020). In the present study, a significantly higher seropositivity was observed in sows than in younger animals (fattening pigs). Age is the most widely discussed variable in literature, and its association with *T. gondii* infection has been demonstrated previously (Castillo-Cuenca et al., 2020, 2021; Cavalcante et al., 2008; Silva et al., 2003).

Thus, it is necessary to implement a sanitary management plan that reduces the exposure of pigs to the protozoan throughout the production chain. This includes providing treated water for the herds and preventing cats from accessing enclosures, facilities, and places of animal feed storage on the production line.

In this study, the use of internal isolation fences on pig farms protected the animals against exposure to *T. gondii.* The presence of an internal isolation fence can hinder or prevent the access of rodents and stray as well as wild animals, including felines, to the interior of enclosures where pigs are allocated, and to places where animal feed is stored. In this manner, fences can minimize the risk of *T.gondii* infection in pigs.

The use of a composting chamber on the farm was a protective factor against toxoplasmic infection in swine in our study, agreeing with Rosa et al. (2018) results that indicates that exposed carcasses favor the incidence of vectors and increase the likelihood of infection in the facilities.

There was no significant association between the presence of rodents and *T.gondii* infection in pigs on the farms sampled in this study. However, it is important to note that rodents are reservoirs of *T. gondii* (Dubey, 2009) and have been suggested to play an important role in the direct transmission of the disease on pig farms because of the consumption of infected rodents by pigs (Kijlstra & Jongert, 2008).

*T. gondii* infections in pigs are frequently asymptomatic; however, several cases of clinical disease and even death have been reported. Further, *T. gondii* infection is associated with reproductive failure in sows, and is characterized by abortion, fetal mummification, stillbirth, and neonatal mortality (Basso et al., 2015). In this study, the presence of reproductive disorders in sows were not statistically significant. The association between reproductive disorders and *T. gondii* infection has been reported (Damriyasa et al., 2004) and can be of considerable clinical and economic importance for agribusiness, rendering this variable an important source of investigation.

## Conclusions

This is the first report on *T. gondii* infection in pigs on farms in Espírito Santo State, Brazil. The seropositivity observed in this study indicates that this zoonotic parasite is widespread in pig breeding farms, with higher prevalence in the Central Serrana microregion. This finding is a public health concern because seropositive pigs can harbor tissue cysts in their meat, thereby representing a tentative zoonotic risk to consumers of raw or undercooked porcine meat or its products. Detection of anti-*T. gondii* antibodies can be very useful for identifying infections in pigs thus serving as an effective control tool for producers. This study also demonstrates that proper sanitary management of pig farms, as the presence of inner fence, is extremely important to reduce the rate of *T. gondii* infection. Finally, the study provides support for the implementation of prevention and control measures against human infections.
